# The Vicious Cross-Talk between Tumor Cells with an EMT Phenotype and Cells of the Immune System

**DOI:** 10.3390/cells8050460

**Published:** 2019-05-15

**Authors:** Elisabetta Romeo, Carmelo Antonio Caserta, Cristiano Rumio, Fabrizio Marcucci

**Affiliations:** 1 Fondazione per la Medicina Solidale, via San Cosimo 8 Pellaro, 89134 Reggio Calabria, Italy; docelisaromeo@gmail.com (E.R.); ace.medicinasolidale@gmail.com (C.A.C.); 2 Department of Pharmacological and Biomolecular Sciences, University of Milan, via Trentacoste 2, 20134 Milan, Italy; cristiano.rumio@unimi.it

**Keywords:** EMT, immune exclusion, immune deviation, cross-talk, cytokines, chemokines, exosomes

## Abstract

Carcinoma cells that undergo an epithelial-mesenchymal transition (EMT) and display a predominantly mesenchymal phenotype (hereafter EMT tumor cells) are associated with immune exclusion and immune deviation in the tumor microenvironment (TME). A large body of evidence has shown that EMT tumor cells and immune cells can reciprocally influence each other, with EMT cells promoting immune exclusion and deviation and immune cells promoting, under certain circumstances, the induction of EMT in tumor cells. This cross-talk between EMT tumor cells and immune cells can occur both between EMT tumor cells and cells of either the native or adaptive immune system. In this article, we review this evidence and the functional consequences of it. We also discuss some recent evidence showing that tumor cells and cells of the immune system respond to similar stimuli, activate the expression of partially overlapping gene sets, and acquire, at least in part, identical functionalities such as migration and invasion. The possible significance of these symmetrical changes in the cross-talk between EMT tumor cells and immune cells is addressed. Eventually, we also discuss possible therapeutic opportunities that may derive from disrupting this cross-talk.

## 1. Introduction

The tumor microenvironment (TME) comprises, in addition to tumor cells, several accessory cell types that contribute to tumor growth and progression. Such contribution is in many cases the result of cross-talk between tumor cells and accessory cells [1]. Angiogenic vascular cells, infiltrating immune cells, and cancer-associated fibroblasts are some of the accessory cells in the TME [1]. Infiltrating immune cells comprise cells of both the native as well as the adaptive immune system. Tumor-associated macrophages (TAMs), subsets of granulocytes, myeloid-derived suppressor cells (MDSCs), dendritic cells (DCs), natural killer (NK) cells, and mast cells are examples of accessory cells of the native immune system, while different T lymphocyte subpopulations, such as CD4^+^ T cells, CD8^+^ T cells, regulatory T cells (Tregs), and B lymphocytes, are examples of cells of the adaptive immune system that can infiltrate the TME.

Tumor cells themselves are not a rigorously homogeneous cell population. In fact, they are endowed with considerable plasticity, allowing them to express different functions and phenotypic markers. An epithelial-mesenchymal transition (EMT) is the main process underlying the heterogeneity of carcinoma cells. Tumor cells undergoing EMT lose properties of epithelial cells, such as the apical–basal axis of polarity and cell–cell adhesion, and acquire properties of mesenchymal cells, such as loose three-dimensional organization and increased motility [2]. EMT, however, is not an all-or-nothing event with tumor cells losing all epithelial markers and acquiring an entirely mesenchymal phenotype. Rather, tumor cells can display hybrid phenotypic and functional states reflecting intermediate tumor cell subpopulations between fully epithelial and fully mesenchymal cells [3].

Tumor cell EMT can occur in different conditions. First, it can occur in response to stressors from the TME, such as hypoxia, a low pH, immune responses, mechanical stress, and antitumor drugs. These responses are largely mediated by growth factors and cytokines such as transforming growth factor (TGF)-β [4]. Second, stressor-promoted epigenetic changes that induce heritable effects allow for retention of the mesenchymal state even when the stressors are no longer present [5]. Third, signaling pathways are activated independently of a stimulus due to the activation of oncogenic mutations or tumor-associated overexpression of pathway components [6]. From a functional point of view, tumor cells undergoing EMT are characterized by increased invasiveness and metastasis formation, resistance to apoptosis and antitumor drugs, and the acquisition of tumor-initiating potential [7].

EMT induced by extracellular stimuli is the result of cross-talk between tumor cells and accessory cells from the TME, including cells from the immune system [4]. EMT, however, can by itself induce phenotypic and functional changes in accessory cells of the TME, including cells of the innate and adaptive immune systems. In the following, we discuss the modalities of the cross-talk between tumor cells and cells of the immune system with regard to the induction of EMT by immune cells and EMT-driven changes in cells of the immune system.

## 2. The Cross-Talk between EMT Tumor Cells and Cells of the Immune System

The cross-talk between EMT tumor cells and cells of the immune system favors, by and large, the induction of EMT in tumor cells by immune cells and the inhibition of antitumor immune responses by tumor cells that have acquired mesenchymal traits as a consequence of an EMT (hereafter referred to as EMT tumor cells). While this is a general rule, there are exceptions, which will be discussed in the following. Before addressing in detail the main aspects that characterize this cross-talk, we will first discuss the evidence suggesting that the cross-talk between EMT tumor cells and the immune system is relevant in the human setting and of functional significance to overall tumor progression and patient prognosis. This is not a trivial issue, since, for example, it has been questioned over the years whether EMT actually occurs in the human setting and if it is of any pathophysiological significance [8].

### 2.1. Association of EMT and an Immunosuppressive TME in Patients

There is now considerable evidence, derived mainly from immunohistochemical and gene expression studies, suggesting that tumors enriched in EMT markers are associated with an immunosuppressive TME and a negative prognosis. In the following, we provide some examples of these studies.

Gene expression clustering studies in ovarian cancer have shown that the mesenchymal subtype, enriched in an EMT-related gene signature, has a worse prognosis and survival compared to other subtypes [9]. In this mesenchymal subtype, a decreased number of CD8^+^ tumor-infiltrating lymphocytes (TILs) were detected, suggesting an association between EMT tumor cells and exclusion of these immune cells from the TME. Similarly, in a study conducted in non-small cell lung cancer (NSCLC), EMT markers were associated with reduced tumor infiltration of CD4^+^ and CD8^+^ T cells, increased expression of immunosuppressive cytokines such as interleukin (IL)-10 and TGF-β, as well as overexpression of inhibitory immune checkpoint molecules such as cytotoxic T-lymphocyte antigen (CTLA)-4 and T-cell immunoglobulin and mucin domain-containing (TIM)-3 [10].

In an immunohistochemistry study performed in patients with gastric cancer, a high expression of EMT traits, the infiltration of TAMs, and the expression of TGF-β1 were associated with a negative prognosis [11]. In a study of lung adenocarcinoma [12], EMT markers were associated with enhanced tumor infiltration of CD4^+^Foxp3^+^ Tregs and upregulation of inhibitory immune checkpoint molecules such as programmed cell death (PD)-ligand (L) 1, PD-L2, TIM-3, B7-H3, and CTLA-4. B7-H3 was identified as a negative prognostic marker for NSCLC. Similarly, a genomic and proteomic analysis based on a tumor cell EMT signature conducted across almost 2000 different tumors [13] revealed a strong association between EMT and markers of inhibited or exhausted immune responses. Thus, a high expression of inhibitory immune checkpoint molecules such as PD-1, PD-L1, CTLA-4, OX40L, and PD-L2 was observed in tumors with the most mesenchymal EMT scores. An association between EMT tumor cells (mesenchymal and hybrid epithelial-mesenchymal phenotypes) and increased numbers of infiltrating PD-1^+^ cells was also observed in another study in patients with adenocarcinoma of the lung [14]. A very strong association was found between PD-L1 expression and the claudin-low subset of breast cancer, which is characterized by a high EMT score [15].

Overall, these studies allowed for the identification of two different scenarios: first, an association between EMT and a reduced infiltration of immune cells (i.e., a predominantly immune-excluded TME); and second, an association between EMT and the infiltration of suppressive or exhausted immune cells (i.e., a predominantly immune-deviated TME). In both cases, however, the conclusions are similar: EMT markers associate with a TME that has inhibitory effects (through immune exclusion or deviation) on antitumor immune responses.

### 2.2. EMT-Associated Changes in the Immunological Profile of Tumor Cells

EMT tumor cells undergo phenotypic alterations that have significant consequences for the recognition by cells of the native and adaptive immune systems. Both down- and upregulation of cell surface molecules of immunological significance have been described. In general, these changes are accompanied by immune resistance and evasion, but there are exceptions to this rule.

The first class of phenotypic alterations consists of the regulation of factors directly or indirectly involved in immune recognition. Thus, EMT-like alterations in melanoma cells have been reported to reduce the expression of multiple tumor antigens, with a consequent escape from being killed by T cells specific for these antigens [16]. However, when targeting antigens whose expression was unaltered during EMT, the capacity of T cells to kill melanoma cell lines in vitro was unaltered [17]. Similarly, T cell-driven immunoediting of breast tumors in *neu*-transgenic mice led to the emergence of antigen-loss variants that had undergone an EMT [18]. Reduced antigen presentation may depend on the downregulation of components of the antigen processing machinery. Thus, EMT NSCLC cells showed a significantly reduced expression of immunoproteasome components and their regulators [19]. The immunoproteasome generates antigenic peptides that bind to human leukocyte antigen (HLA)-I molecules for recognition by CD8^+^ T cells. Consequently, reduced expression of the immunoproteasome leads to reduced presentation of antigenic peptides. EMT-associated downregulation of molecules involved in the presentation of antigenic peptides can also lead to tumor cells hiding from immune recognition. This has been shown for HLA-I molecules, which were downregulated in epithelial cell lines of different tumors as a result of EMT [20,21], with a consequent reduction in antigen presentation and recognition by CD8^+^ cytotoxic T lymphocytes (CTLs) [20].

Upregulation in tumor cells of inhibitory immune checkpoint molecules of native and adaptive immunity can also occur in tumor cells in response to EMT. These molecules inhibit the onset or the continuation of ongoing antitumor immune responses. EMT has been associated with the upregulation of several inhibitory immune checkpoint molecules, such as PD-L1 [22,23], TIM-3 [24], B7-H3 [25], B7-H1 [26], or CD47 [27]. Upregulation of PD-L1 induced resistance to CTL-mediated killing [28]. Interestingly, some of these immune checkpoint molecules have been shown to act by themselves as EMT inducers and promote the acquisition of tumor-initiating potential [15,20,25,26,29], thereby contributing to the amplification of the EMT process and its associated functionalities [30].

Another broad class of EMT-associated tumor cell alterations consists of the acquisition of resistance to killing by cytotoxic effector cells independently of antigen display on the tumor cell surface. This has been shown for tumor cells overexpressing the EMT transcription factor (TF) brachyury and the consequent upregulation of the transmembrane glycoprotein mucin-1 (MUC-1). Overexpression of MUC-1 led to reduced susceptibility to killing by tumor necrosis-related apoptosis-inducing ligand (TRAIL) and to CTL lysis [31]. Brachyury has also been shown to reduce the susceptibility of tumor cells to cytotoxic lymphocytes (CD8^+^ T lymphocytes and NK cells) due to inefficient caspase-dependent apoptosis, but in the presence of normal levels of HLA-I molecules, antigenic peptides, or the various components of the antigen presentation machinery [32]. In fact, increased resistance to apoptosis is recognized as a hallmark of EMT [2]. Mechanisms other than resistance to apoptosis appear to operate in some other situations. Thus, it has recently been reported that hypoxia-exposed lung adenocarcinoma subclones with a predominant mesenchymal phenotype displayed resistance toward CTLs and NK cells through a mechanism involving defective immune synapse signaling [33]. In human breast adenocarcinoma cells, reduced susceptibility to CTL-mediated lysis depended on upregulation of the stem cell marker Kruppel-like factor-4 (KLF-4) and miR-7 downregulation [34]. Hypoxia-inducible miR-210 inhibited the susceptibility of lung carcinoma and melanoma cells to lysis by CTLs [35]. An interesting study has reported that acquisition of an EMT phenotype by breast cancer cells was associated with morphologic changes, actin cytoskeleton remodeling, and increased resistance to CTL-mediated lysis [36]. Resistant cells exhibited attenuation in the formation of an immunologic synapse with CTLs along with induction of autophagy. Autophagy appeared to be critical to resistance to CTL-mediated lysis because silencing of beclin1 to inhibit autophagy restored susceptibility to CTL-induced lysis. Thus, this article introduces autophagy as another player in EMT-associated resistance to immune effector mechanisms. EMT and autophagy are, in many instances, mutually exclusive mechanisms of tumor cell resistance, but in addition to the present article, other examples of coexistence or sequential acquisition of markers of EMT and autophagy have been described (discussed in Reference [37]).

While the EMT-associated changes of the immunological profile of tumor cells that have been discussed so far lead to the inhibition of antitumor immune responses, there are some significant exceptions. Thus, colon cancer cells undergoing EMT have been shown to undergo upregulation of ligands activating NK cells, as well as downregulation of HLA-I molecules [20,38]. These changes led to reduced recognition of the tumor cells by CD8^+^ CTLs and enhanced recognition by NK cells. Expression of the NK cell ligands, however, was very low in vivo in advanced tumors with invasive properties, and, concomitantly, enhanced infiltration of NK cells was observed [20]. These results suggested that NK cells had eliminated NK cell ligand-expressing tumor cells and had selected variants that were not recognized by NK cells.

Recent results have shown that EMT can directly increase tumor cell susceptibility to NK cells, thereby contributing, at least in part, to the inefficiency of the metastatic process [39]. The depletion of NK cells allowed spontaneous metastasis without affecting primary tumor growth. EMT-induced modulation of E-cadherin and cell adhesion molecule 1 (CADM1) mediated increased susceptibility to NK cytotoxicity. These results are of considerable interest because, contrary to most results published on this issue, they showed that tumor cell EMT could exert antimetastatic effects through enhanced susceptibility to NK cells. At odds with these results, a recent study showed that EMT-like changes in melanoma cells were induced by NK cells and were dependent on the engagement of the activating NK receptors NKp30 or NKG2D and the release of cytokines [40]. Moreover, an EMT-associated upregulation of HLA-I molecules was observed. This favored an escape from NK cell attack given the protective role of HLA-I molecules toward NK cell cytotoxicity and because of the contemporary downregulation of tumor-recognizing activating receptors on NK cells. On the other hand, upregulation of HLA-I molecules was expected to enhance CTL-mediated cytotoxicity toward tumor cells. How these results can be reconciled with the previous ones remains to be established, but may depend, among other things, on the timing of the tumor progression at which the experiments were performed [20]. Table 1 summarizes the findings that have demonstrated EMT-associated changes in the immunological profile of tumor cells.

### 2.3. EMT-Induced Effects on Cells of the Immune System

So far, we have discussed the consequences of EMT-associated alterations to the immunological recognition of tumor cells. An EMT in tumor cells, however, can also have direct effects on cells of the immune system. These effects can be twofold. The first is immune exclusion, i.e., reduced tumor infiltration of immune cells that mediate effective antitumor immune responses. The second is immune deviation, i.e., deviation from an effective antitumor immune response (suppressed and/or inefficient) without necessarily being accompanied by reduced tumor infiltration of cells of the immune system. The different effects can be present at the same time, albeit at varying degrees, with one predominating over the other. Of note, many EMT-induced effects on cells of the immune system are similar to those observed upon activation in tumor cells of oncogenic pathways that are also involved in the induction of EMT [41]. It appears obvious that these two events, i.e., the induction of EMT and immunosuppressive effects in the TME, are intimately linked in a causal relationship through the activation of oncogenic and EMT-promoting pathways in tumor cells.

With regard to immune exclusion, we have already discussed studies showing that EMT is associated with immune exclusion in human cancers [10,12]. A more direct demonstration has been brought in experimental systems showing that EMT tumor cells cause reduced infiltration of immune cells into the TME [22]. On the other hand, a large number of studies have shown that EMT tumor cells can induce enhanced tumor infiltration of deviated immune cells, such as polymorphonuclear MDSCs [42], myeloid cells [43], mast cells [44,45], or natural CD4^+^CD25^−^ Tregs [46]. Pancreatic cancer cells resistant to anti-vascular endothelial growth factor (VEGF) treatment secreted increased levels of proinflammatory factors, which stimulated the recruitment of CD11b^+^ proangiogenic myeloid cells and acted also in an autocrine manner to induce and amplify tumor cell EMT [47]. In other situations, both the exclusion of effector CD8^+^ T lymphocytes and enhanced infiltration of immunosuppressive MDSCs have been demonstrated [48]. Immune deviation through EMT-mediated induction of an immunosuppressive phenotype has been reported for the M2 polarization of macrophages by bladder cancer cell lines [49] and the induction of the immunosuppressive molecule indoleamine 2,3-dioxygenase (IDO) in monocyte-derived macrophages. These IDO-expressing macrophages suppressed T cell proliferation and promoted the expansion of immunosuppressive Tregs [50]. Similarly, mesenchymal-like breast cancer cells were shown to induce activation of macrophages to acquire phenotype and functionalities of immunosuppressive TAMs [51]. EMT tumor cells also induced the generation of immunosuppressive regulatory DCs (DCreg), which induced immunosuppressive CD4^+^Foxp3^+^ Tregs and eventually impaired the induction of antitumor CTLs [52,53]. In an entirely in vitro system where lung, breast, or hepatocellular carcinoma cells were cocultured with T lymphocytes, B lymphocytes, and NK cells, EMT induction in tumor cells led to NK- and T-lymphocyte apoptosis, the inhibition of lymphocyte proliferation, and the stimulation of regulatory T and B cells. IDO was involved at least in part in these effects [54].

In several instances, a bidirectional cross-talk has been demonstrated, with EMT tumor cells inducing immunosuppressive changes in the TME and immune cells inducing further amplification of tumor cell EMT [51]. An interesting example of such a bidirectional cross-talk between EMT tumor cells and cells of the immune system has been brought recently [55]. Overexpression of the extracellular matrix protein secreted protein acidic and rich in cysteine (SPARC) in breast cancer cells reduced their growth rate and induced EMT. This led to the formation of an immunosuppressive TME with increased infiltration of Tregs, mast cells, and MDSCs. On the other hand, inhibition of the suppressive function of MDSCs could revert tumor cell EMT, thereby showing that MDSCs contributed to the induction and/or amplification of tumor cell EMT. In a KRAS^G12D^-driven mouse model of lung cancer [56], tumor cells expressing the EMT transcription factor Snail secreted a soluble mediator, which increased Gr1^+^ neutrophil infiltration and secretion of the chemokine C-X-C motif chemokine ligand (CXCL) 2 by the neutrophils themselves. The neutrophils, on the other hand, favored tumor growth, reduced T cell homing in the tumor, prevented successful anti-PD-1 immunotherapy, and altered angiogenesis, leading to hypoxia and sustained Snail expression in the tumor cells [56]. Bladder cancer cells have been shown to recruit mast cells to the tumor [44]. Recruited mast cells could then enhance bladder cancer cell invasion. Thyroid cancer cells recruited and activated mast cells in the TME [57], which in turn released IL-8, which induced EMT and tumor-initiating features in the thyroid cancer cells [57].

So far, we have discussed the suppressive effects of EMT tumor cells on cells of the immune system. Recently, the consequences of the immunosuppressive effects of EMT tumor cells on epithelial tumor cells have also been described [58]. Mammary tumor cells arising from epithelial tumor cell lines expressed high levels of HLA-I, low levels of PD-L1, and were infiltrated by CD8^+^ T lymphocytes and M1-polarized (antitumor) macrophages. On the other hand, tumors arising from EMT carcinoma cell lines expressed low levels of HLA-I, high levels of PD-L1, and were infiltrated by Tregs, M2-polarized (protumor) macrophages, and exhausted CD8^+^ T cells. Importantly, the EMT tumor cells protected their epithelial counterparts from immune attack.

### 2.4. The Mediators of EMT-Induced Effects on Immune Cells

The effects of tumor cell EMT on cells of the immune system are largely mediated by soluble molecules such as cytokines or chemokines rather than by cell-to-cell contact. Recently, a new player, extracellular vesicles, or exosomes, has joined soluble molecules as a mediator of EMT effects on cells of the immune system.

Several molecules have been shown to directly mediate the effects of EMT tumor cells on cells of the immune system. Examples of these factors are the following: chemokines such as IL-8/CXCL8 [42,59], CXCL1, and CXCL2 [48]; C-C motif chemokine ligand (CCL) 2 [53]; CCL20 [50]; cytokines such as IL-6 [60]; TGF-β [46]; the other member of the TGF-β superfamily bone morphogenetic protein (BMP) 4 [49]; granulocyte–macrophage (GM) colony-stimulating factor (CSF) [51]; other soluble mediators such as thrombospondin-1 [52]; and lipocalin 2 [53]. Exosomes have been shown to promote the polarization of macrophages toward the immunosuppressive M2 phenotype upon engulfment by the cells [61]. Table 2 gives a synoptic view of the mediators of the EMT-induced effects on immune cells and the experimental or clinical settings in which the effects were observed.

### 2.5. Induction of Tumor Cell EMT by Cells of the Immune System

In this and the following section, we discuss the other loop of the cross-talk, i.e., the induction of tumor cell EMT by cells of the immune system. Again, we will first discuss which immune cells promote EMT induction, and following that which factors mediate this induction. Both the cells of the native and adaptive immune systems have been shown to induce EMT: Cells of the native immune system include NK cells [40,62], MDSCs [55,63,64], lipopolysaccharide (LPS)-activated macrophages [65,66], TAMs [67,68,69,70,71,72,73], neutrophils [74], and mast cells [57]; cells of the adaptive immune system include TILs [75], activated T lymphocytes [76], CD4^+^ T lymphocytes [77], CD8^+^ T lymphocytes [78], and B lymphocytes [79]. In some cases, EMT induction in tumor cells has been shown to be accompanied, as one might expect, by the acquisition of tumor-initiating potential [78]. Moreover, the stimulation of immune cells with cell type-specific activators can enhance their potential for inducing EMT in tumor cells. Thus, M2-polarized TAMs induced EMT in pancreatic cancer cells in a manner dependent on the expression of the LPS coreceptor toll-like receptor (TLR) 4 and IL-10 secretion [73]. This induction was strongly enhanced upon LPS stimulation of TLR4. Eventually, as for EMT-induced effects on immune cells, immune cell-induced EMT in tumor cells can also be part of a bidirectional cross-talk. In addition to the examples cited in Section 2.3, in a mouse model of pancreatic cancer, tumor cells converted CD14^+^ peripheral blood monocytes into monocytic MDSCs, which in turn induced tumor cell EMT [80].

### 2.6. The Mediators of the Induction of Tumor Cell EMT by Cells of the Immune System

The induction of tumor cell EMT by cells of the immune system is also mediated by soluble factors or extracellular vesicles. Here, a bewilderingly large number of soluble molecules released by cells of the immune system have been shown to induce tumor cell EMT. Examples of these mediators are the following: cytokines such as interferon (IFN)-γ [40,62,81,82], tumor necrosis factor (TNF)-α [16,40,66,76,82,83,84,85], IL-6 [76,77,86,87,88,89,90,91], IL-17 [92,93], IL-18 [94], TGF-β [21,63,67,71,74,76,95], epidermal growth factor (EGF) [21,63,90], hepatocyte growth factor (HGF) [63], high-mobility group box 1 (HMGB1) [96], macrophage migration inhibitory factor (MIF) [97], B-cell activating factor [79], chemokines such as IL-8/CXCL8 [57,68,98,99], CCL2 [88], CCL18 [51,100], and CCL21 [101]. In one case, a cytokine, CSF-1, was shown to induce a partial EMT in inflammatory breast cancer cells. Partial EMT was characterized by upregulation of the mesenchymal marker vimentin, while expression of the epithelial marker E-cadherin was maintained [102]. This article is of interest, since it is one of the few that has investigated the mechanisms of induction of a partial EMT, where both epithelial and mesenchymal markers coexist.

Different soluble mediators may exert additive/synergistic effects on the induction of tumor cell EMT, as has been demonstrated with TGF-β and TNF-α in lung cancer cells [95]; TNF-α, IL-1β, and IL-6 in renal cell carcinoma (RCC) cells [85]; IL-6 and CCL2 in NSCLC cells [88]; TGF-β, IL-6, and TNF-α in inflammatory breast cancer cells [76]; IL-6 and EGF in ovarian carcinoma cells [90]; and IL-6 and TGF-β in NSCLC cells [103]. In one case, each of two soluble mediators, CCL20 and CXCL8, did not induce EMT by itself, but required the contemporary presence of the other chemokine [104]. EMT induction in tumor cells may also be the result of a sequential induction of soluble mediators. Thus, in RCC cells, TNF-α has been shown to induce EMT through increased expression of the chemokine receptors CXCR2 and CXCR3 and their related ligands [105]. In lung adenocarcinoma cells, TGF-β upregulated CXCR7 expression, which mediated the induction of EMT and the acquisition of tumor-initiating potential [106].

With regard to extracellular vesicles, exosomes derived from TILs have been shown to induce EMT in human esophageal squamous cell carcinoma cells [75].

Table 3 gives a synoptic view of the mediators of the induction of tumor cell EMT by cells of the immune system and the experimental or clinical settings in which the effects were observed.

## 3. Activation of Similar Programs in Response to Similar Stimuli in Tumor Cells and Immune Cells

We have discussed the evidence showing that tumor cells and cells of the immune system can undergo a vicious cross-talk favoring the induction of EMT in tumor cells and immune exclusion and/or deviation. In this section, we discuss some recent results suggesting that tumor cells and immune cells can respond to similar stimuli, activating similar programs leading to EMT in tumor cells and to immune exclusion and/or immune deviation. In the following, we also address the possible implications of these observations.

The fact that similar stimuli can induce both effects appears obvious, since several soluble mediators have been discussed in previous sections as being able to induce both tumor cell EMT and inhibition of antitumor immune responses. Of these, TGF-β is the best known and most deeply investigated of these factors. TGF-β is a potent inducer of EMT in general and of tumor cell EMT in particular [107,108,109]. In addition, it induces multiple effects on cells of the immune system. In general, these effects promote the exclusion of immune cells from the TME and the inhibition of immunological recognition and effector functions. Thus, TGF-β promotes the differentiation of naïve CD4^+^ T cell into Tregs [110], inhibits the differentiation of Th1 cells [111], and prevents the expression of Th2-specific cytokines [112]. In mouse models of metastatic colorectal cancer, it inhibited tumor infiltration by T lymphocytes and differentiation of effector Th1 lymphocytes [113]. TGF-β also inhibits effector functions in NK cells and macrophages and antigen presentation by DCs and macrophages [114], and it promotes upregulation of the EMT transcription factor Snail in human THP-1 macrophages, thereby inducing their M2 polarization [115]. An interesting article has reported that TGF-β induces in EMT tumor cells a cluster of genes that is specifically enriched in monocyte-derived macrophages, mast cells, and myeloid DCs (but less so in other types of immune cells) [116]. This was observed upon long-term treatment of mouse mammary EpRas tumor cells with TGF-β1 for two weeks. It was further shown that this gene cluster was enriched in human breast cancer cell lines displaying an EMT or a Basal B profile and in human breast tumors with EMT and undifferentiated characteristics. These are important results, since they show that a single soluble mediator, the cytokine TGF-β, can induce in tumor cells, along with other modifications typical of EMT, a cluster of genes typical of cells of the native immune system.

In the following, we discuss some other examples of mediators that also act on both tumor cells and cells of the immune system. Estrogen receptor-binding fragment-associated antigen 9 (EBAG9) is an estrogen-responsive gene that was originally identified in breast cancer cells. It is a cancer biomarker and promotes the immune escape of tumor cells. It has been recently shown that prostate cancer-derived extracellular vesicles containing EBAG9 can both promote EMT in prostate cancer cells as well as suppress the cytotoxicity of T lymphocytes [117].

HGF is another cytokine that is involved in EMT induction in tumor cells [63,71]. Expression of the specific HGF receptor Met in the immune system is limited to antigen-presenting cells, including DCs. HGF has been found to play a role in peripheral immune tolerance by directing DC differentiation toward a tolerogenic phenotype. In skin immunity, Met signaling drives the mobilization of DCs by regulating matrix metalloproteinase activities [118]. This is reminiscent of tumor cell mobilization in response to EMT induction by HGF and other mediators [2].

Further examples have shown that similar transcription factors can promote EMT in tumor cells and immunosuppressive changes in cells of the immune system. Thus, zinc finger E-box-binding homeobox (ZEB), an E-box transcriptional repressor involved in the induction of tumor cell EMT, has been shown to negatively regulate the expression of the co-stimulatory molecule CD4 in CD4 single-positive T lymphocytes [119] and to act as a transcriptional repressor of IL-2 by binding to the IL-2 promoter [120] and as a repressor of the immunoglobulin heavy chain enhancer [121].

**Figure 1:**
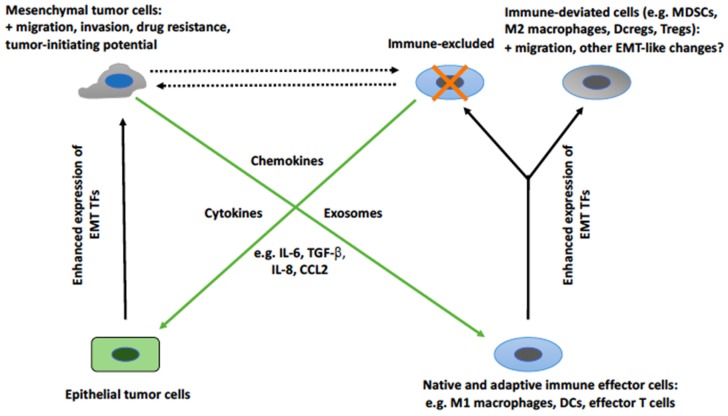
The relationship between changes induced by EMT tumor cells in cells of the immune system and the induction of tumor cell EMT by cells of the immune system. EMT tumor cells induce changes in cells of the immune system that promote their exclusion or deviation toward protumorigenic phenotypes. The mediators of these changes are cytokines, chemokines, or exosomes. At least part of these mediators induce both changes, possibly through the activation of a similar set of EMT transcription factors. Moreover, some of the changes that affect tumor cells and cells of the immune system are similar, such as enhanced migration. The dashed lines indicate an as yet unexplored aspect of this relationship, i.e., the possibility that EMT tumor cells and deviated immune cells regulate each other. Abbreviations: CCL, C-C motif chemokine ligand; DC, dendritic cell; DCreg, regulatory DC; EMT, epithelial-mesenchymal transition; IL, interleukin; MDSC, myeloid-derived suppressor cell; TGF, transforming growth factor; TF, transcription factor; Treg, regulatory T cell.

As already mentioned, overexpression of the other EMT transcription factor, Snail, in human THP-1 macrophages caused their M2 polarization by inhibiting proinflammatory cytokine release and promoting the expression of M2-specific markers [115]. In contrast, Snail knockdown promoted M1 polarization through the upregulation of proinflammatory cytokines. Snail overexpression has also been shown to induce EMT-like functionalities in keratinocytes [122], cells that can act as antigen-presenting cells [123]. These cells acquired an enhanced ability to attract monocytes and to invade a dense interstitial collagen matrix. The maturation of Langerhans cells, a subset of DCs of the skin, was accompanied by downregulation of a set of epithelial genes, including E-cadherin, and by upregulation of the mesenchymal marker N-cadherin (known to facilitate cell migration) [124]. Moreover, the EMT-associated transcription factors ZEB1 and ZEB2 were upregulated in migratory Langerhans cells. These results suggest that these modifications, reminiscent of an EMT, might facilitate the mobilization of Langerhans cells in a manner similar to the acquisition of migratory and invasive properties by EMT tumor cells. Before drawing a premature conclusion that expression of EMT transcription factors in immune cells is always accompanied by immunosuppressive effects, it is fair to note that, in several instances, expression of EMT transcription factors in cells of the immune system has been reported to induce immunostimulating effects [125,126,127]. It is at present unclear what causes an EMT transcription factor to promote immunosuppressive or immunostimulating effects in immune cells. In spite of this limitation, it appears clear that the expression of EMT transcription factors in immune cells can have effects that are not dissimilar to those induced in tumor cells. At present, it is difficult to precisely characterize the functional implications of these observations in the cross-talk between EMT tumor cells and immune cells. Figure 1 represents an attempt to depict and summarize the available knowledge about this cross-talk. It shows that tumor cells responding to soluble mediators released by cells of the immune system undergo an EMT. Vice versa, EMT tumor cells release mediators (soluble mediators or extracellular vesicles) that induce immunosuppressive effects. This cross-talk favors positive amplification loops that favor further recruitment of EMT tumor cells and excluded/deviated immune cells. We have also indicated that EMT cells and immune cells acquire, at least in part, overlapping functionalities such as migration and invasion.

## 4. Some Therapeutic Implications

In this article, we have discussed the cross-talk between tumor cells and cells of the immune system. These two cell populations communicate with each other through soluble mediators and exosomes. Thus, the scenario appears quite simple, but the system appears highly redundant, with many types of immune cells and tumor cells that encompass different stages between a fully epithelial and a fully mesenchymal phenotype. Moreover, a countless number of soluble mediators have been reported to act as go-betweens. It is clear that this scenario poses significant problems from a therapeutic point of view. In fact, if the system is so highly complex and redundant, where is it appropriate to act? On the tumor cell side, on the immune cell side, or on the side of the mediators? From an experimental point of view, many approaches have been pursued, and many successful therapeutic interventions have been reported in the literature in experimental systems. In the following, we discuss a few examples of these approaches.

On the tumor cell side, many approaches have been described that aimed at inhibiting or reversing EMT or depleting EMT tumor cells [2], including those targeting TGF-β (which appears to play a pivotal role among EMT-inducing mediators) [128]. Interestingly, an active immunization approach against the EMT transcription factor brachyury has also been reported [129], and a phase I clinical trial with this vaccine, designated GI-6301, has been initiated in patients with advanced tumors. The inhibition of cytokine or chemokine signaling is another approach to interrupt EMT induction by immune cells or EMT-induced immunosuppressive effects and, for the reasons that were discussed in previous sections, may act on both sides of the cross-talk.

It would go beyond the scope of this article to enter a detailed discussion of the individual therapeutic approaches that have been tested in experimental models in order to interrupt the cross-talk between EMT cells and immune cells. It should be noted, however, that in spite of the multitude of successful experimental interventions that have been reported in the literature, clinical application has lagged behind for most of these. Thus, only a few of these approaches have progressed into a clinic, and positive results in the human setting have been very scarce, if any. This also holds true for the closely related field of anti-CSC candidate drugs [130,131]. The possible reasons for these failures are numerous. One of the reasons that we would like to mention here is that most of the experimental systems that are used for preclinical investigations reflect only to a very limited degree the human situation, which is likely much more complex than most “snapshot” animal models. The situation is likely further complicated by issues such as drug penetration and the onset of drug resistance [132], which certainly play a more important role in the human setting than in small-sized animal models.

Given this problematic background, one is led to ask which might be the most promising approaches to interrupting the cross-talk between EMT cells and immune cells. We envisage three possibilities: first, acting on EMT tumor cells; second, acting on cytokines or chemokines that act as mediators; and third, acting on immune cells. Targeting cytokines or chemokines appears to be very challenging, because this is probably the most redundant component of the cross-talk. Depleting EMT tumor cells appears to be more promising, but this should be combined with approaches aimed at depleting other tumor cells populations, such as differentiated epithelial cells and autophagic tumor cells [37]. The intrinsic phenotypic heterogeneity of EMT tumor cells [3], however, can be a potential problem that is difficult to estimate at present. Immune cells are also difficult to target because of the vast array of immune cell populations that can induce EMT. One possibility is to investigate antibodies targeting inhibitory immune checkpoints (e.g., anti-PD-1, anti-PD-L1, anti-CTLA-4). These antibodies have represented a turning point in cancer therapy [133]. The mechanism of action that is generally ascribed to these drugs is restoring an efficient antitumor immune response [133]. As discussed before, the induction of EMT tumor cells is mainly induced by deviated immune cells (e.g., Tregs, DCregs, MDSCs, M2-polarized TAMs), and it is reasonable to expect that restoring a non-deviated, efficient antitumor immune response may also have favorable consequences on the induction of tumor cell EMT.

## 5. Conclusions

We have discussed the fundamentals of the cross-talk between EMT tumor cells and immune cells. This cross-talk rests, on the one hand, on the induction of EMT in carcinoma cells (or a process bearing similarities with an EMT in noncarcinoma cells, such as melanoma cells) by cells of the immune system with an immune-deviated phenotype. On the other hand, it rests on the induction of immune exclusion and/or immune deviation by EMT tumor cells. While this picture is quite clear, there remain important unresolved questions. First, as already discussed, an EMT in tumor cells is per se a set of phenotypic states that range from a fully epithelial to a fully mesenchymal phenotype. We do not know if the intermediate, hybrid states differ in their capacity to promote immune-excluded or immunosuppressed states. Second, and vice versa, we do not know if the different cell populations with an immune-deviated phenotype differ in their capacity to induce tumor cell EMT or different hybrid states of EMT. Third, the bewildering number of soluble mediators (now also extracellular vesicles) that participate in the cross-talk may also differ in inducing different states of tumor cell EMT and different states of immune exclusion or immunosuppression. Fourth, and most intriguingly, we have seen that tumor cells and immune cells may respond to similar stimuli in a similar manner, i.e., activating similar transcriptional regulators and even similar functional activities. The significance of these “symmetrical” changes is obscure at present. Does it form the basis of a cross-talk between entities that have undergone similar changes and and that may impact tumor progression? In fact, the cross-talk that has been portrayed so far bears on the induction of EMT in differentiated, epithelial tumor cells by immune cells and suppression/deviation in immune cells by EMT tumor cells. Possible reciprocal effects between “full-blown” EMT tumor cells and “full-blown” suppressed/deviated immune cells have not been investigated so far. As can be easily seen from these few traits, much work remains to be done in this field, and many issues remain to be uncovered.

## Figures and Tables

**Table 1:** Epithelial-mesenchymal transition (EMT)-associated changes in the immunological profile of tumor cells.

Type of Alteration	Consequences	References
**Reduced Expression of Tumor Antigens**		
Reduced expression of tumor antigens in melanoma cells	Escape from antigen-specific killing by CTLs	[16]
Emergence of antigen-loss variants in EMT tumor cells from *neu*-transgenic mice	Escape from antigen-specific killing by CTLs	[18]
**Reduced Expression of Antigen-Presenting Molecules**		
Downregulation of HLA class I molecules	Reduced antigen presentation and escape from antigen-specific killing by CTLs.	[20,21]
**Enhanced Expression of Inhibitory Immune Checkpoint Molecules**		
Upregulation of PD-L1, TIM-3, B7-H1, B7-H3, CD47	Downregulation of antitumor immune responses, resistance to killing by CTLs, amplification of tumor cell EMT	[15,20,22,23,24,25,26,27,28,29,30]
**Enhanced Resistance of EMT Tumor Cells to Killing by T Cells**		
Overexpression of MUC-1	Reduced susceptibility to killing by TRAIL and CTLs	[31]
Expression of the EMT TF brachyury, leading to inefficient apoptosis, with normal levels of HLA class I, antigenic peptides, and components of antigen presentation machinery	Reduced susceptibility to killing by CTLs and NK cells	[32]
EMT tumor cells showing defective immune synapse signaling	Resistance to killing by CTLs and NK cells	[33]
Upregulation of KLF-4 and downregulation of miR-7	Reduced susceptibility to killing by CTLs	[34]
Expression of hypoxia-inducible miR-210	Reduced susceptibility to killing by CTLs	[35]
Actin cytoskeleton remodeling, autophagy, and attenuation of an immunological synapse	Autophagy-dependent reduced susceptibility to killing by CTLs	[36]
Downregulation of HL -I and upregulation of ligands activating NK cells	Reduced recognition by CTLs and enhanced recognition by NK cells	[20,38]
Modulation of E-cadherin and CADM1	Increased susceptibility to killing by NK cells and reduced metastasis formation	[39]
EMT-like changes in melanoma cells accompanied by upregulation of HLA class I and downregulation of activating receptors on NK cells	Escape from killing by NK cells	[40]

Abbreviations: CADM, cell adhesion molecule; CTL, cytotoxic T lymphocyte; EMT, epithelial-mesenchymal transition; HLA, human leukocyte antigen; KLF, Kruppel-like factor; MUC, mucin; NK, natural killer; PD-L, programmed cell death ligand; TF, transcription factor; TIM, T-cell immunoglobulin and mucin domain-containing.

**Table 2:** Mediators (soluble mediators or exosomes) of the effects of EMT tumor cells on cells of the immune system.

Mediator	Summary of Experimental Observations.	References
**Cytokines**		
IL-2	IL-2 from cholangiocarcinoma cells with EMT-like features induced generation of CD4^+^ CD25^+^ natural Tregs.	[46]
IL-6	IL-6 induced macrophages to differentiate into M2-polarized macrophages.	[60]
TGF-β	TGF-β from cholangiocarcinoma cells with EMT-like features induced generation of CD4^+^ CD25^+^ natural Tregs.	[46]
BMP-4	Recombinant BMP4 and BMP4-containing conditioned media from bladder cancer cell lines promoted monocyte/macrophage polarization toward an M2 phenotype.	[49]
GM-CSF	GM-CSF from mesenchymal-like breast cancer cells (BT-549, MDA-MB-436, and MDA-MB-231) promoted the acquisition of a TAM-like phenotype by macrophages.	[51]
**Chemokines**		
IL-8/CXCL8	IL-8 from claudin-low TNBC cells induced recruitment of PMN-MDSCs in vitro and in vivo, as determined through neutralization experiments with mAb HuMax-IL8.	[42]
CXCL1, CXCL2	CXCL1 and CXCL2 from mouse ovarian cancer cells promoted tumor infiltration of MDSCs, as determined by knockdown of EMT transcription factor Snail.	[48]
CCL2	CCL2 derived from various tumor cell lines induced, in cooperation with Lipocalin-2, DCregs, which in turn induced Tregs, and finally impaired the induction of tumor-specific CTLs.	[53]
CCL20	CCL20 derived from EMT hepatoma cells induced IDO in monocyte-derived macrophages, which in turn suppressed T-cell proliferations and promoted the expansion of Tregs.	[50]
**Other Soluble Mediators**		
Thrombospondin-1	Snail-transduced melanoma cells with EMT features produced thrombospondin-1, which induced Tregs and impaired DCs in vitro and in vivo.	[52]
Lipocalin-2	CCL2 derived from various tumor cell lines induced, in cooperation with Lipocalin-2, DCregs, which in turn induced Tregs and finally impaired the induction of tumor-specific CTLs.	[53]
**Exosomes**		
	Snail-expressing EMT human head and neck cancer cells activated the transcription of miR-21 to produce tumor-derived exosomes, which were engulfed by CD14^+^ human monocytes, suppressing the expression of M1 markers and increasing that of M2 markers.	[61]

Abbreviations: BMP, bone morphogenetic protein; CCL, C-C motif chemokine ligand; CTL, cytotoxic T lymphocyte; CXCL, C-X-C motif chemokine ligand; DCreg, regulatory dendritic cell; GM-CSF, granulocyte–macrophage colony-stimulating factor; ICAM, intercellular adhesion molecule; IDO, indoleamine 2,3-dioxygenase; IL, interleukin; MDSC, myeloid-derived suppressor cell; miR-21, micro-RNA; PAI, plasminogen activator inhibitor; PMN, polymorphonuclear; TAM, tumor-associated macrophage; TGF, transforming growth factor; TNBC, triple-negative breast cancer; Treg, regulatory T cell.

**Table 3:** Mediators (soluble mediators or exosomes) of the induction of EMT by cells of the immune system.

Mediator	Summary of Experimental Observations.	References
**Cytokines**		
IFN-γ	IFN-γ and other cytokines mediated NK cell-induced increased malignancy of melanoma cells;	[40,61,80,81]
	IFN-γ mediated NK cell-induced EMT and HCC formation in hepatocytes from HBsAg transgenic mice;	
	IFN-γ induced EMT in prostate and papillary thyroid cancer cells.	
TNF-α	TNF-α and other cytokines mediated NK cell-induced increased malignancy of melanoma cells;	[40,75,81,82,83,84]
	TNF-α, with other cytokines, induced EMT in IBC cells in a manner similar to soluble factors from activated T cells;	
	TNF-α induced EMT in papillary thyroid cancer and HCC cells;	
	TNF-α induced EMT in HCC cells, with synergistic induction in combination with IL-1β and IL-6;	
	TNF-α derived from macrophages induced tumor cell EMT.	
TGF-β	TGF-β induced HLA class I downregulation and EMT in prostate cancer cells;	[21,62,66,70,73,75,95]
	TGF-β mediated, with other cytokines, EMT-like changes induced by MDSCs in melanoma cells;	
	TGF-β mediated EMT of HCCs induced by TAMs;	
	TGF-β was the main mediator of EMT induced in teratocarcinoma cells by macrophage-conditioned medium;	
	TGF-β mediated EMT in lung adenocarcinoma cells induced by polymorphonuclear neutrophils;	
	TGF-β, with other cytokines, induced EMT in IBC cells in a manner similar to soluble factors from activated T cells;	
	TGF-β induced EMT in lung adenocarcinoma cells, with synergistic effects with other cytokines from a macrophage cell line.	
EGF	EGF induced HLA-I downregulation and EMT in prostate cancer cells;	[21,62,89]
	EGF mediated, with other cytokines, EMT-like changes induced by MDSCs in melanoma cells;	
	EGF induced EMT and enhanced the migration of ovarian carcinoma cells.	
HGF	HGF mediated, with other cytokines, EMT-like changes induced by MDSCs in melanoma cells.	[62]
IL-6	IL-6, with other cytokines, induced EMT in IBC cells in a manner similar to soluble factors from activated T cells;	[75,76,85,86,87,88,89,90]
	IL-6 from activated T cells induced EMT in premalignant pancreatic cells;	
	IL-6 induced EMT in breast cancer, CRC, NSCLC, cervical carcinoma, and ovarian carcinoma cells;	
IL-17	IL-6 induced EMT in prostate cancer cell lines.	[92,93]
	IL-17 induced EMT and enhanced migration and invasion in nasopharyngeal carcinoma cell lines.	
IL-18	IL-18 induced EMT in lung adenocarcinoma cells.	[94]
HMGB1	HMGB1 induced EMT in CRC cells.	[96]
MIF	MIF induced EMT in pancreatic cancer cells.	[97]
BAF	BAF induced EMT in pancreatic cancer cells.	[78]
**Chemokines**		
IL-8	IL-8 mediated mast cell-induced EMT in human thyroid cancer cells;	[57,67,91,98]
	IL-8 mediated macrophage-induced EMT in HCC cells;	
	IL-8 induced EMT and migration in human ovarian cancer cells;	
	review of IL-8 as a mediator between inflammation and tumor cell EMT.	
CCL2	CCL2 enhanced IL-6 induced EMT and invasion of NSCLC cells.	[87]
CCL18	CCL18 from TAM induced EMT in breast cancer cells;	[51,99]
	CCL18 induced EMT and the invasion and migration of pancreatic cancer cells.	
CCL21	CCL21 induced EMT and metastasis in CD133^+^ pancreatic cancer CSCs.	[100]
**Exosomes**		
	Exosomes from TILs induced EMT in human esophageal squamous cell carcinoma cells.	[74]

Abbreviations: BAF, B-cell activating factor; CCL, C-C motif chemokine ligand; CRC, colorectal carcinoma; CSC, cancer stem-like cell; EMT, epithelial-mesenchymal transition; HBsAg, hepatitis B surface antigen; HCC, hepatocellular carcinoma; HGF, hepatocyte growth factor; HLA, human leukocyte antigen; HMGB, high-mobility group box; IBC, inflammatory breast cancer; IFN, interferon; IL, interleukin; MDSC, myeloid-derived suppressor cell; MIF, macrophage migration inhibitory factor; NK, natural killer; NSCLC, non-small cell lung cancer; TAM, tumor-associated macrophage; TGF, transforming growth factor; TIL, tumor-infiltrating lymphocyte; TNF, tumor necrosis factor.
